# Protein Kinase Inhibitors and Oxidative Stress Modulate In Vivo Phosphorylation of *Trypanosoma cruzi* DNA Polymerase β

**DOI:** 10.3390/pharmaceutics18030385

**Published:** 2026-03-20

**Authors:** Edio Maldonado, Matías Oyarce, Paz Canobra, Emilia Rojas, Fabiola Urbina, Julio C. Tapia, Lilian Jara, Vicente J. Miralles, Christian Castillo, Aldo Solari

**Affiliations:** 1 Núcleo Interdisciplinario de Biología y Genética (NiBG), Instituto de Ciencias Biomédicas (ICBM), Facultad de Medicina, Universidad de Chile, Santiago 8380453, Chile; matias.oyarce@ug.uchile.cl (M.O.); paz.canobra@ug.uchile.cl (P.C.); emiliarojas@ug.uchile.cl (E.R.); fabi.urbina1516@gmail.com (F.U.); jtapiapineda@uchile.cl (J.C.T.); ljara@uchile.cl (L.J.); ccastillor@uchile.cl (C.C.); 2 Departamento de Bioquímica y Biología Molecular, Universidad de Valencia, 46110 Valencia, Spain; vicente.j.miralles@uv.es

**Keywords:** *T. cruzi*, protein kinases, protein kinase inhibitors, oxidative stress, tyrosine phosphorylation

## Abstract

**Background/Objectives**: Protein kinases play crucial roles in signal transduction pathways that regulate growth and differentiation in *Trypanosoma cruzi*. These protein kinases are attractive targets to develop new drugs to treat Chagas disease. **Methods:** We used several protein kinase inhibitors targeting the p38 MAPK, MEK, and ERK pathways to evaluate their effects on the in vivo phosphorylation status of *T. cruzi* proteins, particularly DNA polymerase beta (TcPolβ). We also used Genistein, a protein tyrosine kinase inhibitor, to assess its effects on global protein phosphorylation and TcPolβ phosphorylation. Also, we investigated the effect of oxidative stress on global tyrosine phosphorylation. Finally, we determined the phosphorylation sites on TcPolβ by the protein kinases TcPKC2 and TcWee570 in vitro. **Results:** p38 MAPK and MEK protein kinase inhibitors inhibited approximately 50% of the Ser/Thr phosphorylation of TcPolβ. Genistein inhibited both Ser/Thr and Tyr phosphorylation of several polypeptides in epimastigotes. Oxidative stress increases global Tyr phosphorylation by about twofold and also TcPolβ phosphorylation. TcPKC2 and TcWee570 were able to phosphorylate TcPolβ at both Ser/Thr and Tyr residues. **Conclusions:** Small-molecule protein kinase inhibitors can affect the phosphorylation status of TcPolβ in vivo. Since Genistein can inhibit both Ser/Thr and Tyr protein phosphorylation, and TcPKC2 and TcWee570 can phosphorylate both Ser/Thr and Tyr residues, it suggests the existence of dual protein kinases in *T. cruzi*. However, this possibility must be further studied.

## 1. Introduction

The flagellated protozoan *Trypanosoma cruzi* is the etiologic agent of Chagas disease, also known as American trypanosomiasis. This disease is classified as a Neglected Tropical Disease and is endemic to Latin America [1]. It is estimated that 6–8 million people are infected with *T. cruzi*, and 75 million are at risk of infection. Annually, 30,000 new cases are reported, and 10,000 Chagas disease-related deaths occur annually [1,2] due to migratory movements from endemic countries. Chagas disease has become widespread in several non-endemic countries, including Europe, the USA, Australia, and Japan, among others [1,2]. Triatomine vectors frequently transmit Chagas disease. However, blood transfusion, organ transplantation, or congenital transmission through the placenta has gained relevance with human migrations, and it is a challenge for both endemic and non-endemic countries [1].

The life cycle of the *T. cruzi* parasite is complex and intricate. It involves replicative and infective stages in both triatomine vectors and mammalian hosts. The trypomastigotes circulating in the host blood are transformed into the insect intestine to replicating epimastigotes, which can change into metacyclic trypomastigotes in the distal portion of an insect’s intestine [3,4]. These metacyclic trypomastigotes are excreted as insect amastigotes, which enter the host’s circulation as trypomastigotes and replicate intracellularly as amastigotes. This intricate life cycle is a key factor in the parasite’s ability to survive and proliferate.

*T. cruzi*, with its digenetic life cycle alternating between insect vectors and mammalian hosts, must navigate a myriad of environmental conditions to adapt and survive. The parasite’s main environmental challenges include temperature, pH, nutrient availability, ionic composition, osmolarity, oxidative stress, host immune response, contact with host cells and tissues, and intracellular life [5,6,7]. One of the primary strategies for the parasite to adapt and survive in these environments is through cell differentiation, which involves profound changes within the parasite, altering the expression of surface antigens and intracellular components [5,6]. Adaptation to these changes and cell differentiation are mainly mediated by signal transduction pathways that coordinate cellular responses to the new environmental milieu [5,6,7]. Some of these changes must be rapid to enable quick adaptation to new environmental settings. All these processes can be triggered very rapidly by signal transduction pathways. Protein kinases play a crucial role in phosphorylating protein substrates, thereby altering their function. In contrast, protein phosphatases can dephosphorylate target proteins, thereby terminating signal transduction [5]. In trypanosomatids, distinctive protein kinases that control cell cycle progression, cell differentiation, and stress responses have been identified [8]. However, the signal transduction process through reversible protein phosphorylation remains poorly understood in trypanosomatids. Notably, trypanosomatids do not regulate gene expression at the transcriptional level, unlike higher eukaryotes [7].

Protein kinases play a crucial role in signal transduction in eukaryotic cells, including *T. cruzi*. Since there are structural differences between the protein kinases of *T. cruzi* and those of the host, it is feasible to design protein kinase inhibitors that target the parasite’s kinases without affecting the host. These inhibitors could be used as chemotherapeutic agents to treat Chagas disease.

The DNA polymerase β in *T. cruzi* (TcPolβ) is a 403 amino acid phosphoprotein at the kinetoplast (the unique mitochondrion in trypanosomatids), which plays a pivotal role in kDNA repair and kDNA replication [9,10,11]. The significance of TcPolβ cannot be overstated, as it plays a crucial role in kDNA repair and replication, thereby ensuring the parasite’s survival and proliferation. TcPolβ comprises three domains: the first N-terminal lyase domain (L); the second polymerase domain, which is composed of three subdomains, namely, the DNA-binding (D subdomain), the catalytic subdomain (C subdomain), and the nucleotide-binding subdomain (N subdomain) [9,11]; and a third domain located at the C-terminus which is named C-terminal variable domain (CTVD). Due to its phosphorylation state, TcPolβ can be found in two forms in cell-free extracts [10], the L and H forms. The light form (L) is dephosphorylated, whereas the heavy form (H) is phosphorylated in several Ser/Thr, and Tyr residues [12]. The H-TcPolβ form is more active in DNA synthesis, whereas the L form is less active. It has been demonstrated that oxidative stress can increase the levels of the H-TcPolβ form in epimastigotes [12]. Moreover, TcPolβ can be phosphorylated in vitro by various protein kinases, including AUK, CK1, CK2, PKC, and Wee570 [12,13]. Phosphorylation of TcPolβ potentiates the DNA synthesis activity. Recently, we identified the phosphorylation sites on TcPolβ by AUK1, CK1, CK2, and PKC1 using mass spectrometry (MS), revealing that CK1 and PKC1 can phosphorylate several Thr, Ser, and Tyr residues [13]. Notably, phorbol esters can stimulate PKC1 both in vivo and in vitro, thereby augmenting the phosphorylation status of TcPolβ [13].

Signal transduction pathways must play key roles in *T. cruzi* cell growth and differentiation. In this regard, we believe that TcPolβ plays a crucial role in proliferation and that its activity is modulated by both phosphorylation and dephosphorylation. However, much remains to be learned about the expression pattern and phosphorylation status of TcPolβ throughout parasite proliferation. To understand the molecular mechanisms underlying this regulation, we defined three research goals for this work: first, the effect of eukaryotic protein kinase inhibitors on the in vivo phosphorylation status of TcPolβ; second, the effect of oxidative stress on protein phosphorylation; and third, the identification of the in vitro phosphorylation sites on TcPolβ by protein kinases TcPKC2 and TcWee570. To this end, we have used several well-characterized protein kinase inhibitors in vivo, including those targeting the p38 MAPK, ERK, and MEK pathways, as well as the protein tyrosine kinase inhibitor Genistein, to evaluate the phosphorylation status of proteins in epimastigotes, particularly TcPolβ. Since oxidative stress affects TcPolβ phosphorylation, we also evaluated its effects on protein phosphorylation and on TcPolβ in epimastigotes. Additionally, the in vitro phosphorylation sites on TcPolβ by TcPKC2 and TcWee570 were determined by MS. This research is crucial for a deeper understanding of TcPolβ and its role in the survival and proliferation of *T. cruzi*. Additionally, it could shed light on rational drug design for treating Chagas disease.

## 2. Materials and Methods

### 2.1. Cell Culture and Treatment of T. cruzi Epimastigotes

Epimastigotes (Y strain) were cultured in Liver Infusion Tryptose (LIT) supplemented with 10% heat-inactivated Fetal Calf Serum (FCS, Capricorn Scientific, Ebsdorfergrund, Germany) [12,14] at 28 °C until reaching mid-log phase and then treated with different protein kinase inhibitors and hydrogen peroxide.

### 2.2. Protein Kinase Inhibitors

Protein kinase inhibitors such as U0126, PD184352, PD98059, SB203580, Genistein, TBB, and Gouml 6983 were purchased from Cell Signaling Technology (Danvers, MA, USA). Epimastigote cells were treated with protein kinase inhibitors for 8 h at 28 °C, then harvested and lysed in lysis buffer. The cell-free extracts were then analyzed by immunoprecipitation and Western blot. To determine the optimal working concentrations of the protein kinase inhibitors, a dose–response curve was performed. The response curve was performed within the previously published concentration range. A working concentration balancing strong inhibition of TcPolβ phosphorylation with minimal toxic effects to avoid off-target effects.

### 2.3. Protein Expression and Purification

Genes encoding proteins TcPKC2 (*PWV04907*), TcWee570 (*RNF24358*), and TcPolβ (*RNC61524*) were inserted into pET15b (Novagen, Merck KGaA, Darmstadt, Germany) and transformed into *E. coli* BL21 (DE3) cells, and their expression was induced by the addition of 0.5 mM IPTG [12,13]. All recombinant TcPKC2, TcWee570, and TcPolβ proteins were solubilized in guanidium hydrochloride from the inclusion bodies, renatured, and purified according to references [12,13,15] in NTA-Ni-agarose resin. Protein concentrations were determined using the Bio-Rad protein assay Kit (Hercules, CA, USA) with BSA as the standard. The purity of recombinant proteins was determined using PAGE-SDS and followed by colloidal Coomassie Blue G-250 staining (Invitrogen, ThermoFisher Scientific, Waltham, MA, USA).

### 2.4. In Vitro Phosphorylation Assays

The in vitro phosphorylation assays were performed as described in references [12,13] and included 1 mM ATP (Promega Corporation, Madison, WI, USA). As depicted in each Figure legend, quantities and combinations of the different recombinant proteins were added.

### 2.5. TcPolβ Immunoprecipitation

PureProteome Protein A magnetic beads (Merck Millipore, Catalog LSKMAGA02) were washed with PBS, incubated with rabbit anti-TcPolβ serum at 2 mg of IgG per mL of settled resin on a rocking platform, and cross-linked to the beads using Dimethyl Pimelidate according to [16]. The resin was blocked and extensively washed to remove all non-crosslinked antibodies and stored in 100 mM Tris-HCl, pH 8.0, at 4 °C until use. Twenty microliters of beads were mixed with 200 µL (400 µg) of protein extracts from *T. cruzi* epimastigotes prepared in lysis buffer (50 mM Tris-HCl, pH 8.0, 300 mM NaCl, 5 mM EDTA, 0.01% *w/v* SDS, 0.05% *w/v* sodium deoxycholate, 0.1% *v/v* NP-40, 0.1% *v/v* Triton X-100, and 5 mM of each orthovanadate, sodium fluoride and glycerol-phosphate as phosphatase inhibitors. The lysis buffer was supplemented with 1 tablet of complete protease inhibitor (cOmplete EDTA-free, Catalog 1183617000, MilliporeSigma, Burlington, MA, USA) per 10 mL of solution. Each protein extract was adjusted to the same protein concentration, although there were no differences between them when the same number of parasites was used for extraction. The protein and beads were mixed on a rocking platform for 2 h at room temperature, washed three times with lysis buffer (1.4 mL each wash), and then with 1 mL of PBS. After that, the beads were resuspended in 80 μL of 1X SDS sample buffer and heated at 100 °C for 10 min. Typically, 20 μL of the sample is loaded onto a 10% PAGE-SDS for Western blot analysis.

### 2.6. Western Blot Analysis

TcPolβ was phosphorylated using the appropriate reaction buffer [13] in a 20 μL final volume. Phosphorylated TcPolβ was separated in a 10% PAGE-SDS gel and transferred to Immobilon-E membranes, which were blocked in bovine serum albumin (BSA) at 4% *w/v* plus 1% *w/v* of gelatin in TTBS (50 mM Tris-HCl, 150 mM NaCl, 0.1% *v/v* Tween20, pH 8.0) for 2 h at room temperature. After, the membranes were incubated overnight with anti-phosphor Ser/Thr monoclonal antibody (ThermoFisher Scientific, Catalog MA5-38234) at a dilution of 1/10,000 in 4% *w/v* of BSA in TTBS buffer or anti-phosphorTyr monoclonal antibody (Cell Signaling Technologies, Catalog 9411, Danvers, MA, USA) at a dilution of 1/1000 in 4% *w/v* of BSA in TTBS at 4 °C with gentle rocking. After this step, the membranes were washed with TTBS and incubated with anti-mouse IgG conjugated to horseradish peroxidase (HRP) at a dilution of 1/10,000 (Cell Signaling Technologies, Catalog 7076) in TTBS for 45 min at room temperature. Once incubation was complete, the membranes were washed three times with TTBS (each wash lasting 10 min), then overlaid with SuperSignal West Atto Ultimate Sensitivity Substrate (Catalog A38555, Thermo Fisher Scientific) and exposed to X-ray film. The films were developed, dried, and scanned. The Western blot for TcPolβ was performed as described in reference [10]. Images were quantified using ImageJ 1.52a software (image.net).

### 2.7. TcPolβ Phosphosite Identification

TcPolβ (20 µg, about 500 pmol) was phosphorylated with 100 pmol TcPKC2 or 100 pmol TcWee570 in 200 µL reaction volume with 1 mM ATP in the adequate reaction buffer described in reference [13]. Phosphorylated TcPolβ was separated using a 9% PAGE-SDS gel and visualized by colloidal Coomassie Blue G-250 stain. The band corresponding to the TcPolβ polypeptide was excised from the gel and sent for phosphopeptide and phosphorylation site analysis at the Proteomic Analysis Center, University of Valencia, Spain. Briefly, the phosphorylated TcPolβ in the gel slices was treated with trypsin. Then the peptides were extracted from the gel slices, enriched by TiSO4 chromatography, separated by nanoLC, and identified by MS/MS analysis. The raw MS files were analyzed and searched against the RNC61524 (TcPolβ) protein reference sequence using MaxQuant (1.6.2.6). The identified peptides and phosphorylation sites were listed in an Excel sheet and then mapped to the TcPolβ reference protein sequence.

### 2.8. Statistical Analysis

Results were expressed as the mean *±* standard deviation (SD). Experiments were repeated at least three times. The area of interest in the film was measured using ImageJ 1.52a software. The same rectangular area was scanned for each condition. Graphics were constructed in Microsoft Excel 2019 using calculations based on the previous value, as determined by ImageJ 1.52a. Differences between means in all data presented in this work were analyzed for statistical significance using Student’s *t*-tests. Significance was considered when *p* < 0.05.

## 3. Results

### 3.1. Protein Kinase Inhibitors Can Affect In Vivo TcPolβ Phosphorylation

**Figure 1:**
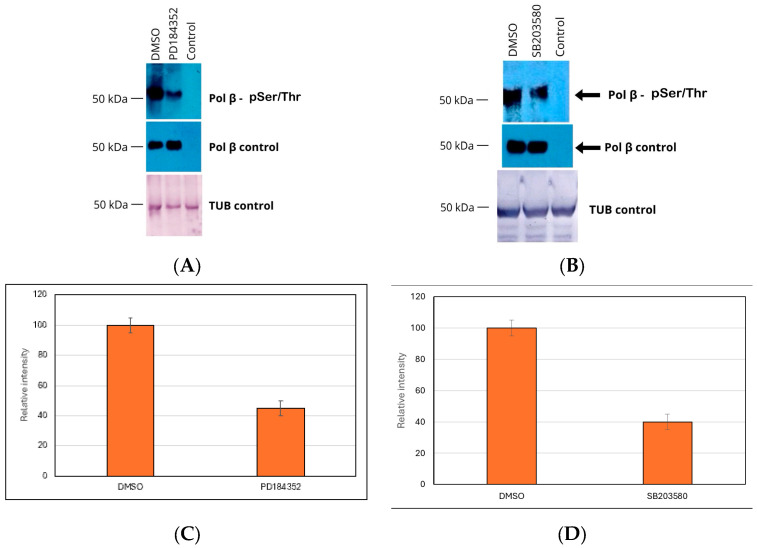
MEK and p38 MAPK Ser/Thr kinases phosphorylate in vivo to TcPolβ. Epimastigote cells were grown in LIT media and treated as indicated at the top of the figure with DMSO or 1 μM of protein kinase inhibitors (PD 184352 or SB203580) for 8 h at 28 degrees Celsius. The cells were then harvested, and protein extracts were prepared, immunoprecipitated with anti-TcPolβ antibodies cross-linked to protein A agarose beads and analyzed by Western blot as described in Materials and Methods. The lane labeled “control” was an extract immunoprecipitated with preimmune IgG cross-linked to protein A agarose beads. (**A**) Analysis of the effect of the MEK protein kinase inhibitor PD184352. DMSO treatment as a control. The upper panel shows Western blot analysis using an anti-pSer/Thr monoclonal antibody; the middle panel shows Western blot analysis using an anti-TcPolβ antibody; and the lower panel shows a Western blot of the tubulin loading control for the protein extracts used in each experiment. The control represents an immunoprecipitation using preimmune IgG crosslinked to magnetic beads. (**B**) Analysis of the effect of the p38 MAPK protein kinase inhibitor SB203580. DMSO was used as a control. Panels depicted are similar to those in A. (**C**) Quantification of Figure 1A, upper panel, representing the mean +/− SD of four (*n* = 4) independent experiments. The *p* < 0.05 between the DMSO- and PD184352-treated groups indicates statistical significance. (**D**). Quantification of Figure 1B. upper panel, representing the mean +/− SD of three (*n* = 3) independent experiments. The *p* < 0.05 between the DMSO- and SB203580-treated groups indicates statistical significance.

The TcPolβ is phosphorylated both in vitro and in vivo [13,17]. In cell-free extracts, it can exist in two forms: H (phosphorylated) and L (non-phosphorylated) [11,17]. To identify protein kinases that can in vivo affect the phosphorylation of TcPolβ, we treated *T. cruzi* epimastigotes with several well-characterized protein kinase inhibitors, as indicated in the figure legends. These protein kinase inhibitors used included U0126 (MEK), PD184352 (MEK), PD98059 (MEK1), SB203580 (p38 MAPK), TBB (CK2), and Gouml 6983 (PKC). We chose to target the MAPK signaling pathway since it is a central regulator of cell signaling and links extracellular stimuli to intracellular responses. The MAPK pathway regulates proliferation, differentiation, apoptosis, and stress responses. Cell-free extracts were prepared from the treated epimastigotes and immunoprecipitated with anti-TcPolβ antibodies. The immunoprecipitants were then analyzed by Western blot using anti-pSer/Thr antibodies. The results showed that the protein kinase inhibitors PD184352 and SB203580 inhibited the in vivo phosphorylation of TcPolβ (Figure 1A and 1B, respectively). The inhibition was greater than 50%, as shown in Figure 1C,D. However, the total levels of TcPolβ did not change, indicating that only its phosphorylation levels were affected by the protein kinase inhibitors (see middle panel). Both cell-free extracts had the same amount of protein, as indicated by the tubulin loading control (lower panel). The remaining protein kinase inhibitors, namely Gouml 6983, TBB, PD98059, and U0126, did not affect TcPolβ phosphorylation at the concentrations used in the experiments.

More than 50% inhibition of TcPolβ phosphorylation by a protein kinase inhibitor is biologically significant, as TcPolβ is a phosphoprotein involved in kDNA replication and repair, processes that might be key regulatory mechanisms for parasite survival and proliferation. Moreover, the MAPK pathway might be essential for other important biological processes, including cell cycle regulation, proliferation, DNA repair, and stress response signaling.

### 3.2. T. cruzi Epimastigote Cell-Free Extracts Contain Phosphoproteins

**Figure 2:**
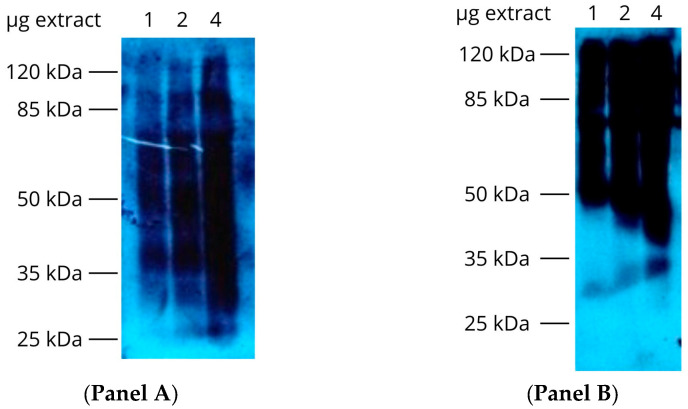
Epimastigote cell-free extracts contain phosphoproteins. Protein extracts were prepared from epimastigote cells grown in LIT media and analyzed by Western blot with anti-phospho antibodies. In (**Panel A**), 1, 2, and 4 μg were analyzed by Western blot using anti-pTyr monoclonal antibody. In (**Panel B**), the same amount of proteins as in panel A was analyzed and reacted with anti-pSer/pThr monoclonal antibody.

To determine whether epimastigotes contain detectable phosphoproteins, we prepared cell-free extracts from *T. cruzi* epimastigotes. As described in Materials and Methods, phosphoproteins were detected using anti-pTyr and anti-pSer/pThr antibodies. Different amounts of protein were tested for each antibody, and the results are shown in Figure 2. Both antibodies react with several polypeptides in the cell-free extract, indicating the presence of low- to high-molecular-weight proteins phosphorylated on Tyr, Ser, and Thr residues in epimastigote protein extracts.

### 3.3. The Protein Tyrosine Kinase Inhibitor Genistein Can In Vivo Inhibit Tyr and Also Ser/Thr Phosphorylation

**Figure 3:**
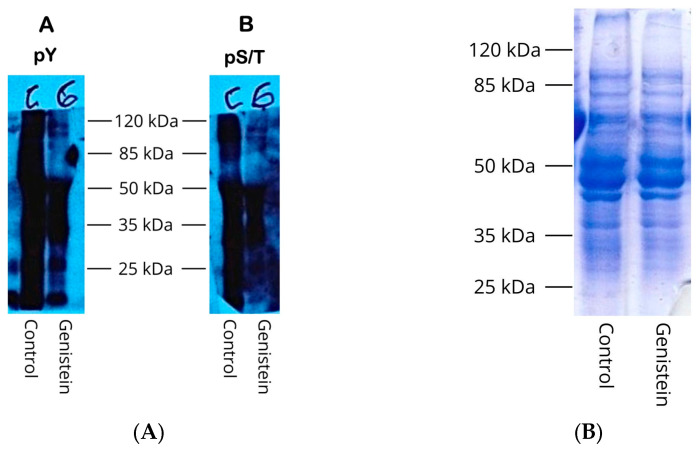
Genistein inhibits in vivo protein phosphorylation in *T. cruzi* epimastigotes. Epimastigote cells were grown in LIT medium, treated with 75 μg/mL Genistein for 8 h at 28 °C. DMSO treatment was used as a control. (**A**) Protein extracts were prepared, and 5 μg of each protein extract was analyzed by Western blot, as indicated at the bottom of the figure with anti-pTyr (pY, panel A) and anti-pSer/pThr (pS/T, panel B) monoclonal antibodies. (**B**) 5 μg of each protein extract was analyzed in a 10% SDS-PAGE, followed by Coomassie Blue R-250 staining.

Typical tyrosine protein kinases have not been found in trypanosomatids, although Tyr phosphorylation has been detected in the bloodstream and procyclical forms of *T. brucei* and *T. cruzi* epimastigotes [18,19,20,21,22]. Genistein is a protein tyrosine kinase inhibitor in higher eukaryotic cells, and it can inhibit the proliferation of procyclical *T. brucei* and *T. cruzi* epimastigote cells [20,22]. To determine whether Genistein could inhibit Tyr phosphorylation, we treated *T. cruzi* epimastigotes with the inhibitor and measured Tyr phosphorylation in cell-free extracts by Western blot using anti-phosphor antibodies. The results are presented in Figure 3, which shows that Genistein can largely inhibit Tyr phosphorylation (panel A) but, interestingly, Ser/Thr phosphorylation as well (panel B). In the presence of Genistein, the epimastigotes did not divide. The whole protein pattern of the Genistein-treated epimastigotes did not change significantly, as evidenced by SDS-PAGE (Figure 3B). In addition, tubulin levels remained constant in both experimental and control groups of parasites. These findings suggest that the protein Tyr-kinase inhibitor, Genistein, indeed interferes with Tyr phosphorylation, but unexpectedly also affects Ser/Thr phosphorylation in *T. cruzi* epimastigotes.

### 3.4. TcPolβ Phosphorylation Is Affected by Genistein

**Figure 4:**
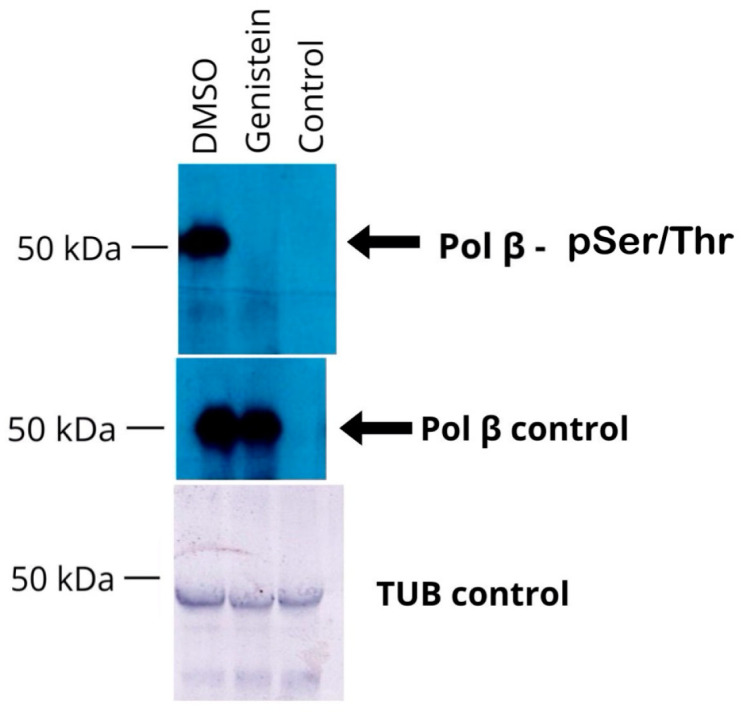
Genistein affects in vivo TcPolβ phosphorylation. Protein extracts were obtained as described in Figure 3, immunoprecipitated, and analyzed by Western blot as described in Materials and Methods. The upper panel represents a Western blot analysis with anti-pSer/pThr monoclonal antibody, as well as with anti-TcPolβ antibody (middle panel). The lower panel shows the tubulin loading control of each protein extract.

Next, we investigated whether specifically TcPolβ Ser/Thr phosphorylation is affected by Genistein in *T. cruzi*. Cell-free extracts from Genistein-treated epimastigotes were immunoprecipitated with anti-TcPolβ antibodies and analyzed for Ser/Thr phosphorylation by Western blot. The results presented in Figure 4 indicate that TcPolβ Ser/Thr phosphorylation is largely inhibited by Genistein, while it is detected in the DMSO control. These results are not due to the lack of TcPolβ in the Genistein-treated epimastigotes, since it is detected at the same levels as in the control using anti-TcPolβ antibodies (Figure 4, middle panel). These results indicate that Genistein can modulate in vivo TcPolβ Ser/Thr phosphorylation in *T. cruzi* epimastigotes.

### 3.5. Hydrogen Peroxide Stimulates Protein Tyrosine Phosphorylation

**Figure 5:**
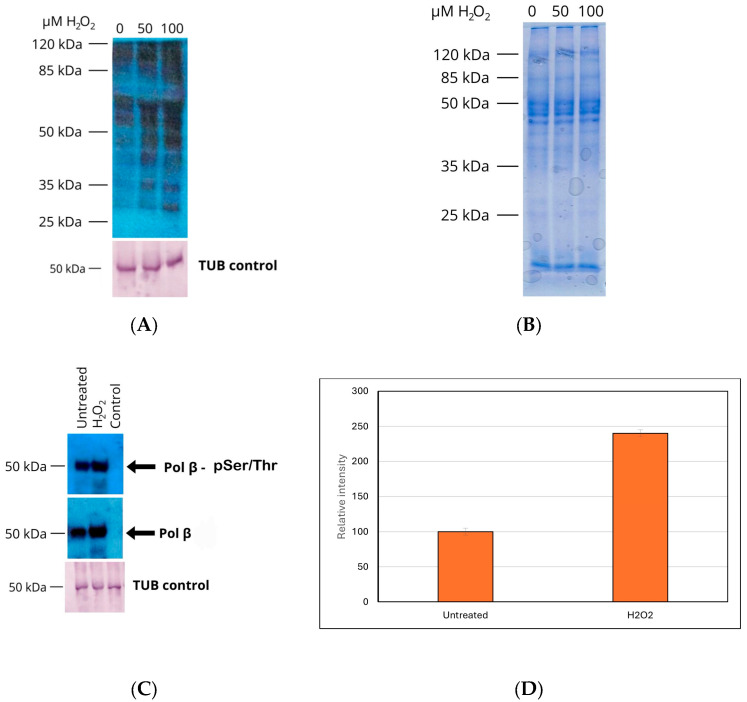
Hydrogen peroxide increases in vivo protein tyrosine phosphorylation. Epimastigotes were grown in LIT medium and incubated with hydrogen peroxide for 8 h at 28 °C. Afterward, protein extracts were prepared and analyzed by Western blot using anti-phospho monoclonal antibodies. (**A**) Epimastigote cultures were treated with 0, 50, and 100 μM of hydrogen peroxide as indicated at the top of the figure. The protein extracts (5 μg) were analyzed by Western blot using an anti-pTyr monoclonal antibody (upper panel) and a tubulin loading control (lower panel). (**B**) A 10% SDS-PAGE was used to analyze 5 μg of protein from epimastigote cell extracts treated with 0, 50, and 100 μM hydrogen peroxide, as indicated at the top of the figure. The gel was Coomassie Blue R-250-stained. (**C**) Protein extracts from epimastigote cell cultures, untreated or treated with 100 μM hydrogen peroxide as indicated at the top of the figure, were immunoprecipitated with anti-TcPolβ crosslinked to protein agarose beads as described in Materials and Methods and analyzed by Western blot with anti-pSer/Thr monoclonal antibody (upper panel) or anti-TcPolβ antibody (middle panel). The lower panel shows a Western blot analysis of tubulin, a loading control for each protein extract. The lane labeled control was an extract immunoprecipitated with preimmune IgG crosslinked to protein A agarose beads (**D**) Quantification of A, upper panel. representing the mean +/− SD of three (*n* = 3) independent experiments. The *p* < 0.05 between the untreated and the hydrogen peroxide-treated groups indicates statistical significance.

We have previously demonstrated that hydrogen peroxide can stimulate TcPolβ phosphorylation in *T. cruzi* epimastigotes [17]. Additionally, hydrogen peroxide can stimulate Tyr phosphorylation of proteins in procyclic *T. brucei* [21]. Therefore, we investigated whether hydrogen peroxide could increase Tyr phosphorylation in *T. cruzi* epimastigotes and the phosphorylation levels of TcPolβ, as detected by immunoprecipitation followed by Western blot with an anti-phosphor antibody. The results presented in Figure 5A indicate that hydrogen peroxide can stimulate overall Tyr phosphorylation in epimastigotes compared with the untreated control. Tubulin levels remain unchanged with treatment (Figure 5A, lower panel). The total protein pattern is not affected by the hydrogen peroxide treatment, as detected by SDS-PAGE (Figure 5B). On the other hand, the level of Ser/Thr phosphorylation of TcPolβ increases about two-fold by the hydrogen peroxide treatment, as observed in Figure 5C (upper panel) and Figure 5D. The total levels of TcPolβ also increase by the hydrogen peroxide treatment (Figure 5C, middle panel). The tubulin levels are not affected by the hydrogen peroxide treatment, as shown in Figure 5C (lower panel). These results suggest that hydrogen peroxide stimulates overall protein Tyr phosphorylation and also the Ser/Thr phosphorylation of TcPolβ in *T. cruzi* epimastigotes.

### 3.6. TcPolβ Is In Vitro Phosphorylated by TcPKC2 and TcWee570

**Figure 6:**
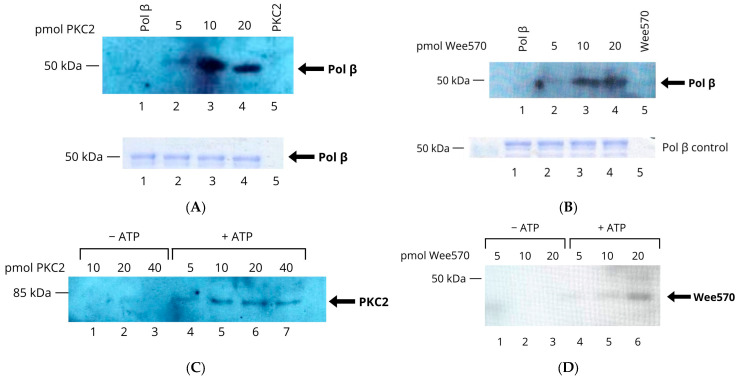
TcPolβ is in vitro phosphorylated by TcPKC2 and TcWee570. Recombinant TcPolβ (200 ng) was incubated with 5, 10, and 20 pMol of recombinant TcPKC2 or TcWee570 in the presence of ATP. The reactions were separated in 10% SDS-PAGE and analyzed by Western blot with anti-pSer/Thr monoclonal antibodies. (**A**) phosphorylation by TcPKC2 (lanes 2–4). (**B**) Phosphorylation by TcWee570 (lanes 2.4). The lower panel shows the TcPolβ loading analyzed by SDS-PAGE and Coomassie Blue R-250 staining. Lane 1 in each experiment contains only TcPolβ, and lane 5 contains only the protein kinase. (**C**) TcPKC2 gets auto-phosphorylated. 10, 20, and 40 pmol of recombinant TcPKC2 were incubated in the absence of ATP (lanes 1.3) and 5, 10, 20, and 40 pmol in the presence of ATP (lanes 4–7). The reactions were analyzed by Western blot using anti-pSer/Thr monoclonal antibodies. (**D**) Autophosphorylation of TcWee570. In lanes 1–3, 5, 10, and 20 pMol of TcWee570 were analyzed in the absence of ATP, whereas in lanes 4–6, the same amount as in lanes 1–3 was analyzed in the presence of ATP. The reactions were analyzed by Western blot using anti-pSer/Thr antibodies.

In an earlier study, we reported that TcPolβ can be phosphorylated in vitro by the TcPKC2 and Wee570 protein kinases [12]. We wanted to confirm these results further using Western blot analysis with an anti-pSer/pThr antibody. The results are presented in Figure 6A, which shows that TcPKC2 can phosphorylate TcPolβ in vitro, as evidenced by an antibody that specifically recognizes phosphorylated TcPolβ (lanes 2–4); however, the antibody does not react with unphosphorylated TcPolβ (lane 1). All the reactions contain the same amount of TcPolβ (Figure 6A, lanes 1–4 (lower panel). On the other hand, TcWee570 can also phosphorylate TcPolβ (Figure 6B, lanes 2–4), and the antibody does not react with the unphosphorylated enzyme. The loading control indicates that all reactions contain the same amount of TcPolβ (Figure 6B, lanes 1–4, lower panel). These results suggest that both TcPKC2 and TcWee570 can in vitro phosphorylate TcPolβ.

During the process of TcPolβ phosphorylation by TcPKC2 and TcWee570, we observed that these kinases become auto-phosphorylated, as they react with the anti-pSer/pThr antibody in the absence of a TcPolβ substrate. We decided to investigate this by performing reactions both in the absence and presence of ATP. The results of these experiments are shown in Figure 6C,D. TcPKC2 is auto-phosphorylated when ATP is present (Figure 6C, lanes 4–7) since it reacts with the anti-pSer/pThr antibody, but no reaction was obtained in the absence of ATP (lanes 1–3). Likewise, TcWee570 gets auto-phosphorylated in the presence of ATP (Figure 6D, lanes 4–6); however, there is no auto-phosphorylation in the absence of ATP (lanes 1–3). From these experiments, we can conclude that TcPKC2 and TcWee570 can be in vitro auto-phosphorylated.

### 3.7. Determination of the In Vitro Phosphorylation Sites on TcPolβ by TcPKC2 and TcWee570 Protein Kinases

**Figure 7:**
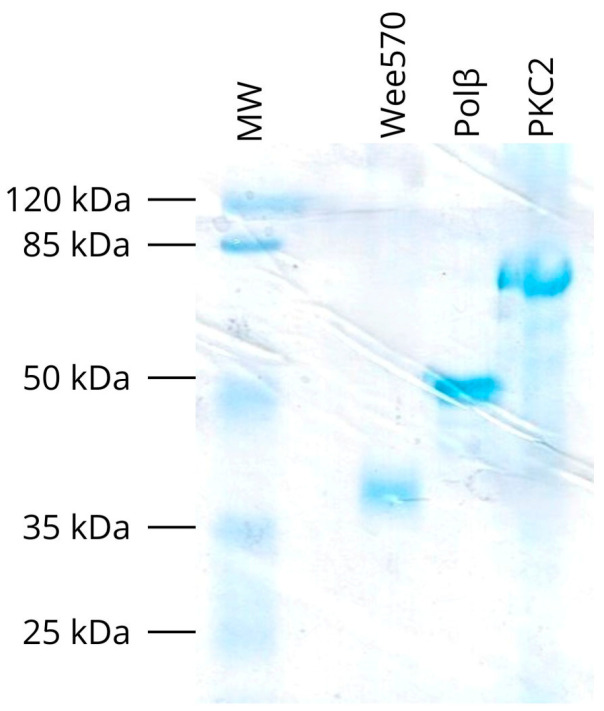
SDS-PAGE analysis. The purity of the different recombinant proteins (200 ng) was analyzed in an 8% SDS-PAGE, followed by colloidal Coomassie Blue G-250 staining.

Despite earlier studies [12] and our previous results showing that TcPolβ is an in vitro substrate for TcPKC2 and TcWee570 kinases, the exact phosphorylation sites on TcPolβ remain unknown. To determine the precise phosphorylation sites by these kinases, we in vitro phosphorylated recombinant TcPolβ with TcPKC2 and TcWee570. All proteins used in this study were at least 90% pure as judged by SDS-PAGE followed by colloidal Coomassie Blue G-250 staining (Figure 7). After phosphorylation of TcPolβ by TcPKC2 or TcWee570 protein kinases, SDS-PAGE separated the proteins, and the phosphorylated TcPolβ was excised from the gel and trypsin-digested. Peptides were recovered, and the phosphopeptides were enriched by TiSO4 chromatography and then subjected to MS/MS detection. As presented in Table 1, several Ser, Thr, and Tyr residues on TcPolβ were found to be phosphorylated by TcPKC2 and TcWee570 protein kinases.

## 4. Discussion

In this work, we report that the eukaryotic protein kinase inhibitors of the MAPK signaling pathway, namely PD184352 and SB203580, can inhibit TcPolβ phosphorylation in vivo. Genistein, a tyrosine protein kinase inhibitor, inhibited both Ser/Thr and Tyr phosphorylation in *T. cruzi* epimastigotes. On the other hand, hydrogen peroxide-induced oxidative stress can stimulate overall protein Tyr phosphorylation in epimastigotes and Ser/Thr phosphorylation of TcPolβ. Also, we demonstrated that the TcPKC2 and TcWee570 protein kinases can in vitro phosphorylate TcPolβ at both Ser/Thr, and Tyr residues. Additionally, these two protein kinases can be autophosphorylated in vitro. These are novel observations and different from our previously published work dealing mainly with in vitro studies on the phosphorylation of TcPolβ by recombinant Ser/Thr protein kinases.

Protein kinases are among the most prominent protein families in eukaryotes, with almost 600 members in mammals [23]. *T. cruzi* has a reduced kinome compared to humans, with 190 genes encoding protein kinases; however, in these parasites, three protein kinase families (GMGC, STE, and NEK) are expanded compared to humans [24]. It is essential to study protein kinases in trypanosomatids, as the kinome differs significantly from that of mammals, including low sequence identity and the presence of unique protein kinases without orthologues. Protein kinases may play key roles in the life cycle and cell cycle of trypanosomatids; however, fewer than 2% of *T. cruzi* protein kinases have been analyzed using loss-of-function approaches [25]. Importantly, protein kinases in trypanosomatids are potential drug targets for treating diseases caused by these parasites [24,26]. Unfortunately, *T. cruzi* lacks RNAi machinery to downregulate genes on a large scale, making it difficult to study the biological functions of these protein kinases.

In this study, we found that *T. cruzi* epimastigotes contain several polypeptides with phosphorylated Ser/Thr and Tyr residues, as determined by Western blot analysis of cell-free extracts. These polypeptides ranged in molecular weight from high to low and appeared to be very abundant. Indeed, Tyr-phosphorylated polypeptides have been detected in procyclic form *T. brucei* cell-free extracts by Western blot using an anti-pTyr antibody [21]. More recently, phosphoproteomic studies in *T. cruzi* epimastigotes have identified 753 phosphoproteins, totaling 2572 phosphorylation sites, of which 2162 (84.1%) were on serine, 384 (14.9%) on threonine, and 26 (1%) on tyrosine [27]. Quantitative proteomics and phosphoproteomics of the *T. cruzi* epimastigote cell cycle have revealed that 597 protein groups and 94 phosphopeptides are regulated, with variation during S-phase of the cell cycle [28]. We believe that the phosphorylated polypeptides detected on the Western blot may regulate various biological processes, including the cell cycle, cell division, DNA replication, transcription, and cell signaling, among others.

Several small, cell-permeable protein kinase inhibitors have been developed in the last few decades. These protein kinase inhibitors exhibit a relatively high degree of specificity for a particular protein kinase, making them helpful in identifying the physiological substrates and cellular functions of these kinases. Importantly, kinase inhibitors are used to treat several diseases, particularly various cancers [29,30,31]. In this work, we used inhibitors targeting the MAPK kinases MEK and p38 MAPK to investigate whether they affect TcPolβ phosphorylation in *T. cruzi* epimastigotes. Significant inhibition of TcPolβ phosphorylation was achieved with the protein kinase inhibitors PD184352 and SB203580, which target the MEK and p38 MAPK pathways, respectively [32,33,34,35]. This indicates that protein kinases from these families, or related families, could, in vivo, phosphorylate or regulate TcPolβ phosphorylation. Indeed, members of these kinase families are encoded in the *T. cruzi* genome [5,24,36,37].

Protein tyrosine-specific phosphorylation plays a crucial role in regulating cell proliferation, growth, differentiation, migration, metabolism, and programmed cell death in metazoans. Moreover, Tyr kinases are involved in all stages of neoplastic development and progression [38,39]. The human genome contains 90 Tyr kinase genes: 58 receptor types, divided into 20 subfamilies, and 32 nonreceptor types, grouped into 10 subfamilies [39]. Trypanosomatid genomes do not contain typical Tyr kinase-encoding genes; however, tyrosine phosphorylation has been documented in *T. brucei*, *T. cruzi*, and *Leishmania* sp. [20,22,40].

Genistein, a mammalian Tyr kinase inhibitor, can inhibit the growth of bloodstream forms of *T. brucei* and epimastigotes of *T. cruzi*, indicating that tyrosine phosphorylation is required for cell proliferation in these parasites [20,22]. Indeed, we detected several proteins whose Tyr-phosphorylation is inhibited by Genistein. Moreover, phosphoproteomic studies have identified several tyrosine-phosphorylated phosphopeptides in *T. cruzi* [27,28].

Surprisingly, in *T. cruzi* epimastigotes, Genistein also inhibited Ser/Thr-phosphorylation to a similar extent as Tyr-phosphorylation. Since *T. cruzi* does not possess typical tyrosine kinases, this result might be explained by the existence of dual specificity protein kinases that can phosphorylate both Ser/Thr and Tyr residues as well. Support for the hypothesis comes from the inhibition of Ser/Thr and Tyr phosphorylation by Genistein. Also, most of TcPolβ phosphorylation occurs on Ser/Thr residues, and its phosphorylation is in vivo inhibited by Genistein, suggesting that dual specificity protein kinases may phosphorylate TcPolβ to regulate its activity. Finally, in this study, we demonstrated that TcPKC2 and TcWee570 can phosphorylate TcPolβ in vitro at Ser/Thr and Tyr residues, acting as dual-specificity protein kinases. Taken together, these results suggest the presence of dual-specificity protein kinases in *T. cruzi*. Indeed, dual specificity protein kinases are present in higher eukaryotes and regulate cell growth, differentiation, apoptosis, and stress responses [41]. For example, MEK1/2 [41] and Myt1, a Wee1-like protein kinase [42], have been shown to function as dual specificity protein kinases and can phosphorylate both Ser/Thr and Tyr residues. Thus, whether a putative TcMEK1-like protein kinase exists in *T. cruzi* and, together with TcWee570, is indeed a dual-specificity protein kinase that can phosphorylate proteins at both Ser/Thr and Tyr residues remains to be investigated.

Dual-specificity protein kinases in *T. cruzi* might contain a unique catalytic site that binds genistein, thereby preventing Ser/Thr and Tyr phosphorylation. This protein kinase inhibitor is an ATP-competitive inhibitor of protein tyrosine kinases. It competes with ATP for binding at the catalytic site of tyrosine kinases, thereby preventing substrate phosphorylation. Genistein has been shown to be a protein tyrosine inhibitor with no effect on Ser/Thr protein kinases [43], but in other studies, it has been reported to directly inhibit Ser/Thr protein kinases, such as PLK1, in cells and in vitro assays [44]. Also, Genistein can modulate the activity of protein Ser/Thr kinases such as MAPK and AKT [45].

Another, less likely, possibility is that Genistein could target an upstream protein tyrosine kinase required to activate protein Ser/Thr kinases in *T. cruzi*. Alternatively, *T. cruzi* might contain atypical protein kinases that phosphorylate both Ser/Thr and Tyr residues and can be targeted by Genistein.

In an earlier study, we demonstrated that hydrogen peroxide-induced oxidative stress can stimulate TcPolβ phosphorylation in *T. cruzi* epimastigotes and trypomastigotes [17]. Furthermore, hydrogen peroxide has been demonstrated to stimulate *T. cruzi* proliferation and promote infection [46]. These observations suggest that oxidative stress impacts the growth of *T. cruzi* cells. The results presented in this work are consistent with this idea, as the hydrogen peroxide treatment of *T. cruzi* epimastigotes stimulates Tyr phosphorylation of several polypeptides. Moreover, hydrogen peroxide stimulates in vivo TcPolβ phosphorylation, as previously reported in our results [17]. Taken altogether, these results strongly suggest that oxidative stress stimulates cell growth in *T. cruzi* epimastigotes, likely through the tyrosine phosphorylation of key proteins involved in cell proliferation. It would be of interest to identify these Tyr-phosphorylated proteins to assess their contribution to cell proliferation in *T. cruzi* and other trypanosomatids.

In *T. cruzi*, elevated ROS levels could directly activate protein kinases, such as MAPKs, or regulate them via redox-sensitive mechanisms, since some protein kinases are regulated by Cys oxidation, altering their phosphorylation activity and, depending on the redox state, either activating or inhibiting their kinase activity [47]. Also, ROS can influence thiol-based regulation by molecules such as trypanothione, which can alter the redox environment and thereby affect kinase activity [47]. In higher eukaryotes, the p38 MAPK signaling pathway is activated by elevated ROS levels and, once activated, phosphorylates and regulates transcription factors and downstream effectors that control cell cycle arrest, apoptosis, senescence, and inflammatory cytokine production [48]. It is most likely that the p38 MAPK pathway plays a role in oxidative stress by ROS in *T. cruzi*; however, this has not yet been demonstrated.

TcPolβ is a DNA polymerase involved in DNA repair and DNA replication of the kDNA in *T. cruzi* [18]. TcPolβ is in vivo phosphorylated at Ser/Thr, and Tyr residues, and in vitro is the target of several protein kinases [12,13]. TcPKC2 and TcWee570 are two protein kinases that in vitro phosphorylate TcPolβ [12]. In this study, we confirmed this observation by using anti-pSer/pThr antibodies and Western blot analysis. Most likely, phosphorylation of TcPolβ by these two protein kinases can affect its activity, since in vitro phosphorylation of TcPolβ by recombinant protein kinases, including Ck1, CK2, PKC1, and AURK, augments TcPolβ’s DNA synthesis activity. Moreover, the in vivo phosphorylated form (form H) of this DNA polymerase is more active in DNA synthesis, while the unphosphorylated form (form L) is less active [15]. It is also probable that these protein kinases can in vivo phosphorylate TcPolβ to regulate the activity of this DNA polymerase.

Notably, TcPKC2 and TcWee570 protein kinases become in vitro autophosphorylated at Ser/Thr residues, suggesting that autophosphorylation may activate or regulate their own catalytic activity.

TcPKC2 and TcWee570 can phosphorylate TcPolβ on several Ser/Thr, and Tyr residues as determined by MS analysis. Notably, Tyr35 is in vivo phosphorylated on TcPolβ [12], and it is in vitro phosphorylated by TcPKC2 and TcWee570, suggesting that these protein kinases can in vivo phosphorylate that site. Several phosphorylated residues overlap with residues in vitro phosphorylated by other protein kinases as well [13]. Additionally, we observed that TcPKC2 preferentially phosphorylates residues on the N- and C-terminal regions of TcPolβ, while TcWee570 preferentially phosphorylates residues at the C-terminal region. Residues in the middle area of TcPolβ are less phosphorylated, probably because they are not easily accessible to the protein kinases.

Studies on trypanosomatid signal transduction pathways and protein kinases are worth pursuing to understand their biological functions, as they are potential drug targets. Since most of the protein kinases from trypanosomatids are sufficiently different from those of mammals, it is feasible to use parasite-specific protein kinase inhibitors to target key parasite kinases to interfere with their life cycle or cell growth. One way to probe differential inhibition is to use an in vitro assay to measure the inhibitor effect on protein kinases from both the host and the parasite.

## Figures and Tables

**Table 1:** Phosphorylated residues on TcPolβ by the indicated protein kinases as determined by MS analysis. Phosphorylated peptides are listed in Supplementary Table S1.

Kinase	Phosphorylated Residues on TcPolβ			
	Y35	T49	S69	T102
Wee570	S193	S275	S299	T333
	S336	T338	T345	S355
	S358	S361	T366	
	Y35	S46	T49	S69
	T102	S185	Y186	S193
TcPKC2	S275	T278	S299	Y302
	Y307	T333	S336	T345
	S355	S358	S361	T366

## Data Availability

The original contributions presented in this study are included in the article/Supplementary Material. Further inquiries can be directed to the corresponding authors.

## Supplementary Materials

The following supporting information can be downloaded at: https://www.mdpi.com/article/10.3390/pharmaceutics18030385/s1. All data supporting reported results and raw data can be requested to the corresponding author Edio Maldonado at emaldona@med.uchile.cl.

## References

[1] De Sousa A.S., Vermeij D., Ramos A.N., Luquetti A.O. (2024). Chagas disease. Lancet.

[2] Sabino E.C., Nunes M.C.P., Blum J., Molina I., Ribeiro A.L.P. (2024). Cardiac involvement in Chagas disease and African trypanosomiasis. Nat. Rev. Cardiol..

[3] Tyler K.M., Olson C.L., Engman D.M. (2003). The life cycle of *Trypanosoma cruzi*. American Trypanosomiasis.

[4] Martín-Escolano J., Marín C., Rosales M.J., Tsaousis A.D., Medina-Carmona E., Martín-Escolano R. (2022). An updated view of the *Trypanosoma cruzi* life cycle: Intervention points for an effective treatment. ACS Infect. Dis..

[5] Huang H. (2011). Signal transduction in *Trypanosoma cruzi*. Adv. Parasitol..

[6] Maeda F.Y., Cortez C., Yoshida N. (2012). Cell signaling during *Trypanosoma cruzi* invasion. Front. Immunol..

[7] Lander N., Chiurillo M.A., Docampo R. (2021). Signaling pathways involved in environmental sensing in *Trypanosoma cruzi*. Mol. Microbiol..

[8] Cayla M., Nievas Y.R., Matthews K.R., Mottram J.C. (2022). Distinguishing functions of trypanosomatid protein kinases. Trends Parasitol..

[9] De Oliveira Lopes D., Schamber-Reis B.L.F., Regis-da-Silva C.G., Rajão M.A., DaRocha W.D., Macedo A.M., Machado C.R. (2008). Biochemical studies with DNA polymerase β and DNA polymerase β-PAK of *Trypanosoma cruzi* suggest involvement in mitochondrial DNA maintenance. DNA Repair.

[10] Maldonado E., Rojas D.A., Moreira-Ramos S., Urbina F., Miralles V.J., Solari A., Venegas J. (2015). Expression, purification, and biochemical characterization of recombinant DNA polymerase β of the *Trypanosoma cruzi* TcI lineage. Parasitol. Res..

[11] Maldonado E., Morales-Pison S., Urbina F., Solari A. (2021). Molecular and functional characteristics of DNA polymerase beta-like enzymes from trypanosomatids. Front. Cell. Infect. Microbiol..

[12] Maldonado E., Rojas D.A., Urbina F., Valenzuela-Pérez L., Castillo C., Solari A. (2022). *Trypanosoma cruzi* DNA polymerase β is phosphorylated in vivo and in vitro by PKC and CK2. Cells.

[13] Maldonado E., Canobra P., Oyarce M., Urbina F., Miralles V.J., Tapia J.C., Solari A. (2024). In vitro identification of phosphorylation sites on TcPolβ by protein kinases TcCK1, TcCK2, TcAUK1, and TcPKC1 in *Trypanosoma cruzi* epimastigotes. Microorganisms.

[14] Castillo C., Carrillo I., Libisch G., Juiz N., Schijman A., Robello C., Kemmerling U. (2018). Host-parasite interaction: Changes in human placental gene expression induced by *Trypanosoma cruzi*. Parasites Vectors.

[15] Maldonado E., Rojas D.A., Urbina F., Solari A. (2021). *T. cruzi* DNA polymerase β is phosphorylated by CK1, CK2 and TcAUK1. PLoS Negl. Trop. Dis..

[16] DeCaprio J., Kohl T.O. (2019). Cross-linking antibodies to beads using dimethyl pimelimidate. Cold Spring Harb. Protoc..

[17] Rojas D.A., Urbina F., Moreira-Ramos S., Castillo C., Kemmerling U., Lapier M., Maldonado E. (2018). Endogenous overexpression of phosphorylated DNA polymerase β under oxidative stress in *Trypanosoma cruzi*. PLoS Negl. Trop. Dis..

[18] Ettari R., Previti S., Maiorana S., Allegra A., Schirmeister T., Grasso S., Zappalà M. (2019). Drug combination studies of curcumin and genistein against rhodesain of *Trypanosoma brucei* rhodesiense. Nat. Prod. Res..

[19] Parsons M., Valentine M., Deans J., Schieven G.L., Ledbetter J.A. (1991). Distinct patterns of tyrosine phosphorylation during the life cycle of *Trypanosoma brucei*. Mol. Biochem. Parasitol..

[20] Wheeler-Alm E.L., Shapiro S.Z. (1992). Evidence of tyrosine kinase activity in the protozoan parasite *Trypanosoma brucei*. J. Protozool..

[21] Nett I.R., Davidson L., Lamont D., Ferguson M.A. (2009). Identification and localization of tyrosine-phosphorylated proteins in *Trypanosoma brucei*. Eukaryot. Cell.

[22] Braga M.V., de Souza W. (2006). Effects of protein kinase and PI3K inhibitors on growth and ultrastructure of *Trypanosoma cruzi*. FEMS Microbiol. Lett..

[23] Zhang H., Cao X., Tang M., Zhong G., Si Y., Li H., Zhao B. (2021). A subcellular map of the human kinome. eLife.

[24] Parsons M., Worthey E.A., Ward P.N., Mottram J.C. (2005). Comparative analysis of the kinomes of pathogenic trypanosomatids. BMC Genom..

[25] Chiurillo M.A. (2025). The challenge of screening essential protein kinases in *Trypanosoma cruzi*. mSphere.

[26] Merritt C., Silva L.E., Tanner A.L., Stuart K., Pollastri M.P. (2014). Kinases as druggable targets in trypanosomatid parasites. Chem. Rev..

[27] Marchini F.K., de Godoy L.M., Rampazzo R.C., Pavoni D.P., Probst C.M., Gnad F., Krieger M.A. (2011). Profiling the *Trypanosoma cruzi* phosphoproteome. PLoS ONE.

[28] Dos Santos A.D.C.M., Melo R.M., Ferreira B.V.G., Pontes A.H., de Lima C.M.R., Fontes W., Ricart C.A.O. (2021). Quantitative proteomics and phosphoproteomics of *Trypanosoma cruzi* epimastigote cell cycle. Biochim. Biophys. Acta Proteins Proteom..

[29] Gross S., Rahal R., Stransky N., Lengauer C., Hoeflich K.P. (2015). Targeting cancer with kinase inhibitors. J. Clin. Investig..

[30] Soltan O.M., Shoman M.E., Abdel-Aziz S.A., Narumi A., Konno H., Abdel-Aziz M. (2021). Molecular hybrids of protein kinase inhibitors for cancer therapy. Eur. J. Med. Chem..

[31] Attwood M.M., Fabbro D., Sokolov A.V., Knapp S., Schiöth H.B. (2021). Trends in kinase drug discovery. Nat. Rev. Drug Discov..

[32] Mattingly R.R., Kraniak J.M., Dilworth J.T., Mathieu P., Bealmear B., Nowak J.E., Reiners J.J. (2006). MAPK/ERK kinase inhibitor PD184352 induces apoptosis. J. Pharmacol. Exp. Ther..

[33] Barančík M., Boháčová V., Kvačkajová J., Hudecová S., Križanová O.G., Breier A. (2001). SB203580 reverses P-glycoprotein-mediated multidrug resistance. Eur. J. Pharm. Sci..

[34] Allen L.F., Sebolt-Leopold J., Meyer M.B. (2003). CI-1040 (PD184352), a targeted signal transduction inhibitor of MEK (MAPKK). Seminars in Oncology.

[35] Paw M., Wnuk D., Nit K., Bobis-Wozowicz S., Szychowski R., Ślusarczyk A., Michalik M. (2021). SB203580—A potent p38 MAPK inhibitor reduces the profibrotic bronchial fibroblasts transition associated with asthma. Int. J. Mol. Sci..

[36] Brumlik M.J., Pandeswara S., Ludwig S.M., Murthy K., Curiel T.J. (2011). Parasite MAP kinases as drug discovery targets. J. Signal Transduct..

[37] Kaur P., Goyal N. (2022). Pathogenic role of MAP kinases in protozoan parasites. Biochimie.

[38] Taddei M.L., Pardella E., Pranzini E., Raugei G., Paoli P. (2020). Tyrosine phosphorylation and cancer metabolism. BBA Rev. Cancer.

[39] Bhanumathy K., Balagopal A., Vizeacoumar F.S., Vizeacoumar F.J., Freywald A., Giambra V. (2021). Protein tyrosine kinases in leukemia. Cancers.

[40] Efstathiou A., Smirlis D. (2021). Leishmania protein kinases as drug targets. Microorganisms.

[41] Roskoski R. (2012). MEK1/2 dual-specificity protein kinases: Structure and regulation. Biochem. Biophys. Res. Commun..

[42] Zhu J.Y., Cuellar R.A., Berndt N., Lee H.E., Olesen S.H., Martin M.P., Schönbrunn E. (2017). Structural basis of wee kinases functionality and inactivation by diverse small molecule inhibitors. J. Med. Chem..

[43] Akiyama T., Ishida J., Nakagawa S., Ogawara H., Watanabe S.I., Itoh N., Fukami Y. (1987). Genistein, a specific inhibitor of tyrosine-specific protein kinases. J. Biol. Chem..

[44] Shin S.B., Woo S.U., Chin Y.W., Jang Y.J., Yim H. (2017). Sensitivity of TP53-mutated cancer cells to the phytoestrogen genistein is associated with direct inhibition of Plk1 activity. J. Cell. Physiol..

[45] Kim S.H., Kim S.H., Kim Y.B., Jeon Y.T., Lee S.C., Song Y.S. (2009). Genistein inhibits cell growth by modulating various mitogen-activated protein kinases and AKT in cervical cancer cells. Ann. N. Y. Acad. Sci..

[46] Paiva C.N., Medei E., Bozza M.T. (2018). ROS and *Trypanosoma cruzi* infection. PLoS Pathog..

[47] Machado-Silva A., Cerqueira P.G., Grazielle-Silva V., Gadelha F.R., de Figueiredo Peloso E., Teixeira S.M.R., Machado C.R. (2016). How *Trypanosoma cruzi* deals with oxidative stress: Antioxidant defence and DNA repair pathways. Mutat. Res./Rev. Mutat. Res..

[48] Schieber M., Chandel N.S. (2014). ROS function in redox signaling and oxidative stress. Curr. Biol..

