# Supplementary material for: Protein Kinase Inhibitors and Oxidative Stress Modulate In Vivo Phosphorylation of Trypanosoma cruzi DNA Polymerase β
Source: Pharmaceutics. 2026 Mar 20;18(3):385. doi: 10.3390/pharmaceutics18030385 (PMC13030474; doi:10.3390/pharmaceutics18030385)
Supplement: Supplementary file 1 [file pharmaceutics-18-00385-s001.zip › Leyenda.Table S1.Suplementaria. Pharmaceutics.pdf]

Supplementary Table S1. A list of phosphorylated peptides of TcPolβ by protein kinases TcPKC2 and TcWee570. Both protein kinases can phosphorylate several Ser/Thr and Tyr residues. In the combined sites in the Excel sheet, column F represents phosphorylation sites on TcPolβ by TcPKC2, and column G represents phosphorylation by TcWee570. The number 1 means that the protein kinase phosphorylates the site, while the number 0 means that the kinase does not phosphorylate that site. Column E shows the identified peptide, and the lowercase letter indicates the phosphorylated residue.
